# Surgical versus conservative treatment of odontoid fractures in the elderly: A randomized controlled clinical study (SCORE)

**DOI:** 10.1371/journal.pone.0337999

**Published:** 2025-12-23

**Authors:** Arthur Wagner, Carolin Albrecht, Sophie Dittmer, Silvia Egert-Schwender, Victoria Kehl, Rita Stichling, Gisela Klatt, Barbara Ettinger, Nils Hecht, Peter Vajkoczy, Alexander Wengert, Frank Kandziora, Nicolas Heinz von der Höh, Christoph-Eckhard Heyde, Philipp Hartung, Marcus Richter, Maximilian Lenz, Peer Eysel, Andreas Badke, Christian Blume, Hans Clusmann, Johannes Walter, Sandro Krieg, Petros Evangelou, Ehab Shiban, Marc Dreimann, Oliver Gembruch, Ulrich Sure, Christoph Bettag, Veit Rohde, Alexander Disch, Biniam Bekele, Yu-Mi Ryang, Andreas Kramer, Florian Ringel, Johannes Wach, Erdem Güresir, Bernhard Meyer, Maria Wostrack

**Affiliations:** 1 Department of Neurosurgery, University Hospital, Technical University Munich, Munich, Germany; 2 Münchner Studienzentrum, School of Medicine and Health, Technical University Munich, Munich, Germany; 3 Osteoporose Selbsthilfegruppen Dachverband e.V., Gotha, Germany; 4 Bundesselbsthilfeverband für Osteoporose e.V., Wendelstein, Germany; 5 Bundesselbsthilfeverband für Osteoporose e.V., Landesverband Bayern, Kitzingen, Germany; 6 Department of Neurosurgery, Charité University Hospital, Berlin, Germany; 7 Department of Spine Surgery, BG Hospital Frankfurt, Frankfurt, Germany; 8 Department of Orthopaedic, Trauma and Plastic Surgery, University Hospital Leipzig, Leipzig, Germany; 9 Spine Center, St.-Josefs-Hospital, Wiesbaden, Germany; 10 Department of Orthopedics and Traumatology, University Hospital Cologne, Cologne, Germany; 11 Department of Spine Surgery, BG Hospital Tübingen, Tübingen, Germany; 12 Department of Neurosurgery, RWTH Aachen University Hospital, Aachen, Germany; 13 Department of Neurosurgery, Heidelberg University Hospital, Heidelberg, Germany; 14 Department of Neurosurgery, Medical University Lausitz Carl Thiem, Cottbus, Germany; 15 Spine Center, Orthopaedic Hospital Markgröningen, Markgröningen, Germany; 16 Department of Neurosurgery, University Hospital Essen, Essen, Germany; 17 Department of Neurosurgery, University Medical Center Göttingen, Göttingen, Germany; 18 Department of Spine Surgery, University Hospital Carl Gustav Carus Dresden, Dresden, Germany; 19 Department of Neurosurgery, Helios Hospital Berlin-Buch, Berlin, Germany; 20 Department of Neurosurgery, University Hospital of Munich LMU, Ludwig-Maximilians-University Munich, Munich, Germany; 21 Department of Neurosurgery, University of Leipzig Medical Center, Leipzig, Germany; PLOS: Public Library of Science, UNITED KINGDOM OF GREAT BRITAIN AND NORTHERN IRELAND

## Abstract

**Background:**

Odontoid fractures of the second cervical vertebra commonly affect elderly patients due to osteoporosis and low-energy trauma. Treatment is controversial, with prolonged cervical collar immobilization risking non-union and complications, and surgical C1–C2 stabilization involving higher upfront surgical risks. High-level evidence from randomized controlled trials to guide optimal treatment decisions is lacking. The SCORE study aims to determine whether surgical stabilization is non-inferior to conservative collar management in maintaining functional independence for elderly patients with unstable odontoid fractures.

**Methods:**

SCORE is a multicenter, parallel-group, randomized controlled non-inferiority trial enrolling 322 patients aged ≥70 years with acute (≤2 weeks) unstable odontoid Type II, III, or atypical fractures. Participants will be randomized 1:1, stratified by center, to receive surgical stabilization via posterior C1–C2 fixation or conservative management with a rigid cervical collar. The primary outcome measure is the change in Barthel Index (BI) from baseline to 12 weeks. Secondary outcomes include quality of life (EQ-5D), neck pain (Visual Analog Scale, VAS), neck disability (Neck Disability Index, NDI), radiographic fusion, treatment compliance, cross-over rates to surgery, and incidence of adverse and serious adverse events up to 6 months. Follow-ups will take place at 12 weeks and 6 months post-injury, with an additional visit at approximately 2 weeks post-surgery for surgical patients. Analysis will use mixed models for repeated measures, targeting 90% power to detect non-inferiority within a 5-point margin on the BI (one-sided α = 0.025), accounting for 15% attrition.

**Discussion:**

This trial addresses a critical evidence gap by directly comparing surgical and conservative treatments, aiming to guide clinical decision-making and improve functional outcomes and quality of life in elderly patients.

**Trial registration:**

ClinicalTrials.gov, ID: NCT06961578

## Background and rationale

Odontoid fractures (OF) are among the most common injuries of the spine and are becoming “endemic” in the geriatric population. As opposed to a younger population in which these fractures occur after high velocity trauma and need to be treated differently, it is now clear by evidence that C2 fractures in the elderly population are insufficiency fractures due to osteoporosis [1,2]. OFs occupy a large volume of our health care system in everyday clinical practice. They continue to gain relevance considering the increasing life expectancy of the aging population, which is particularly susceptible to prolonged morbidity, functional disability, and impaired health-related quality of life [3–7]. Controversy exists as to the optimal treatment, weighing potential failure with external collar bracing against a surgical intervention in an elderly patient [8,9]. As of today, no high-class evidence comparing surgery with conservative bracing in the geriatric population exists and guidelines do not offer clear-cut recommendations as to the optimal treatment [10]. In addition, secondary sequelae of the conservative regimen such as insufficient fracture healing, prolonged pain, deep vein thrombosis and even neurological injury are often underreported and misjudged in clinical practice, oftentimes necessitating secondary surgical stabilization in patients with an impaired general condition [3,11]. OFs occur in 25% of all cervical spine injuries in Germany and Western regions, while in patients older than 65 the incidence is double as high, with OF being the most common cervical spine injury [12]. Geriatric patients are predisposed to OF because of osteoporosis and a propensity to fall. Mortality reaches up to 44% at an average of 2 years after suffering an OF [13,14]. Osteoporosis, a poor blood supply of the fracture gap, and age-associated impaired biomechanics lead to disrupted fracture healing, resulting in an increased non-union rate of up to 85% [5,15–18]. Regardless of the applied treatment, fractures in the elderly bear a high risk of poor outcome and mortality due to co-morbidities and immobilization [19]. The primary goal of therapy is to achieve the fastest possible restoration of pre-traumatic mobilization. The current standard of care treatment consists of either collar bracing of the cervical spine for at least 12 weeks or surgical posterior stabilization with a screw-rod-construct [7,15,20–23].

### State of research

One large prospective non-randomized international registry study, INNOVATE (leadership: Leiden University Netherlands), is underway to assess fracture healing and outcome after surgical vs. conservative treatment of OFs in the elderly [24]. According to the interim analysis of INNOVATE (Huybregts et al., Age and Ageing 2024), no outcome differences were observed between surgical and conservative arms [25]. INNOVATE has defined a radiographic parameter as primary outcome, in addition to the NDI, which has been conceived and validated for the general population without specific aspects for elderly patients. Another randomized clinical trial, the Uppsala Study on Odontoid Fracture Treatment (USOFT; leadership: Uppsala University Hospital Sweden) is still recruiting since its registration in 2016. The study group also chose the NDI as primary outcome parameter, they have, however, excluded demented patients and aim for only 50 patients over both surgical and conservative arms for a statistical power of 80% [26].

### Rationale for the project

We designed our clinical study with the BI as primary outcome to focus on the health-related autonomy in the elderly, specifically including demented patients and patients incapable of giving consent. We aim to answer the principal question of how geriatric patients with substantial comorbidity and frailty can return to their prior level of autonomy and activity after suffering an OF [9]. While we concur that the NDI is suitable for use in the general population, we believe the BI is more adequate to cover the special needs of the elderly [19,27–29]. Our choice of the BI as a primary outcome and NDI as a secondary outcome measure has been proposed after consulting with patient representatives and advocacy groups (Bundesselbsthilfeverband für Osteoporose e.V. (BfO), Selbsthilfe Landesverband für Osteoporose Bayern e.V. (LfO) and Osteoporose Selbsthilfegruppen Dachverband e.V. (OSD), who communicated a need for a measure that is more accessible and relevant in everyday practice [30–36]. This, in conjunction with the shortcomings of available literature and lack of current studies, warrants the conduct of this confirmatory clinical study to expand on the preceding retrospective investigations and provide high quality scientific evidence for clinical decision-making. It must be noted that the geriatric population is drastically underrepresented in published literature, since clinical trials generally eschew this population in particular [37].

## Methods

This is a two-arm, prospective, multi-center, open-label, randomised, controlled non-inferiority clinical study in a parallel group design.

The study compares two established standard methods against each other regarding their efficacy in terms of functional status on follow-up. To date, there is no scientific equipoise concerning the treatment options for OFs, these being surgical stabilization or external collar bracing [4,6,13,38,39]. Hence, both arms are considered as interventions: experimental intervention = surgical group undergoing posterior instrumentation, control intervention = conservative group subjected to external collar bracing.

The clinical study complies with the SPIRIT guidelines and has been approved by the local ethics committee (No. 2024-630-S-NP). The study was registered in clinicaltrials.gov (https://clinicaltrials.gov/study/NCT06961578) prior to the inclusion of the first patient.

### Study population

**Fig 1:**
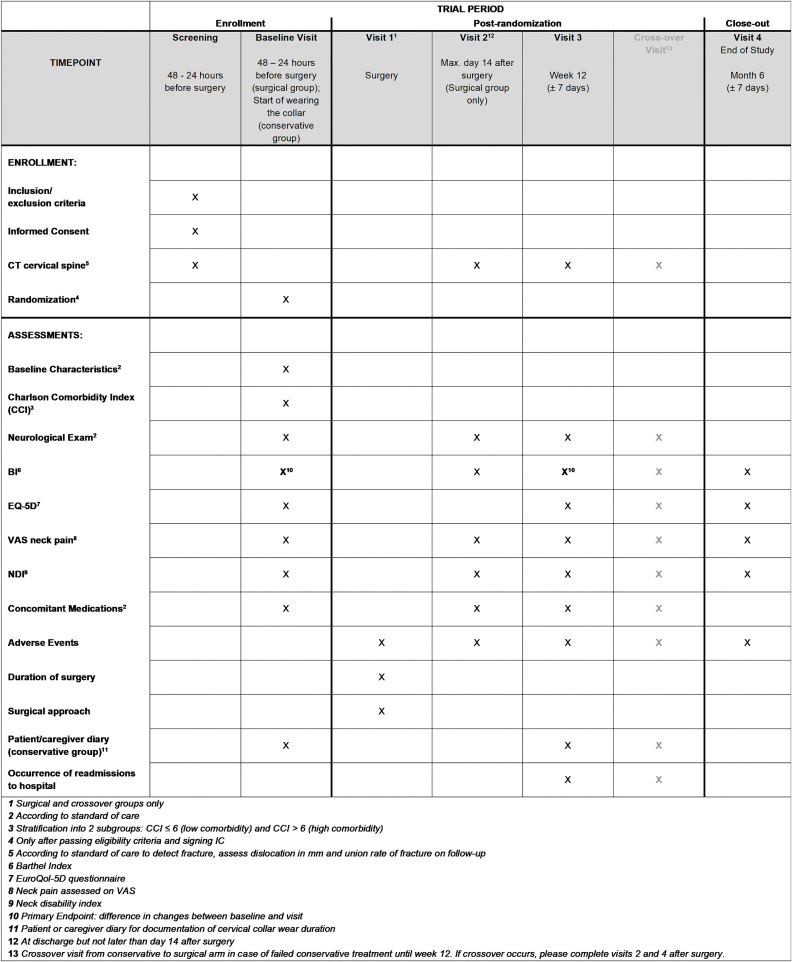
SPIRIT 2025 enrolment flow chart and study timeline [47].

The target population consists of patients with unstable odontoid fractures types II or III as classified by Anderson and d’Alonzo, and atypical C2 fractures. The planned sample size is 322 patients. Fig 1 depicts the study timeline of events.

### Inclusion criteria

- At least 70 years old
- Acute unstable OFs (types II, III as classified by Anderson and d’Alonzo, and atypical)
- Less than two weeks post injury
- Written informed consent

### Exclusion criteria

- Previous treatment for odontoid fracture
- Concomitant fractures of the subaxial cervical spine necessitating surgery
- Significant comorbidity resulting in inoperability of the patient: i.e., ASA score > 4
- Neurological compromise due to displaced fracture

### Measures taken (surgical and conservative treatment)

The surgical intervention is a surgical fracture stabilization with a posterior screw-rod system and represents an established surgical treatment according to standard of care for OFs [3,13,14,20,40,41]. The routine surgery commonly lasts approximately 100 minutes and requires a hospital stay of about 6 days [42,43]. An additional external bracing in the postoperative setting is not required. The surgery is tailored specifically towards geriatric insufficiency fractures of the odontoid, which makes stabilization with a screw-rod-construct from a posterior approach a necessity to achieve adequate stability for this patient population. We deliberately exclude the possibility to conduct an anterior fusion by odontoid screw fixation as described by Böhler, which has been shown to be a biomechanically insufficient treatment particularly in geriatric patients [6,15,23,44,45].

The conservative intervention also aims for immobilization, but via an external collar. A typical detriment of the conservative intervention in everyday practice stems from its absolute reliance on the patient’s compliance to wearing the collar permanently for at least 12 weeks, which considerably challenges this treatment strategy [22]. This compliance problem is highly relevant to the treatment outcome, although data on compliance rates in the elderly are fairly scarce [17,44,46]. We are aware that the regularity of wearing the brace cannot be verified with certainty, and in probably the majority of cases the collar is not worn permanently against the physician`s recommendation due to relevant side effects. Therefore, the adherence to therapy will be assessed via a patient diary in which the hours of wearing the collar will be documented. However, this will reflect the everyday reality and belongs to a real-life representation of the conservative therapy regimen.

Over the course of this study, patients of the conservative group can undergo surgical intervention, if requested by the patient or advised by the treating physician, until week 12. Prior to this crossover surgery, patients are required to attend the crossover visit. After surgery, the patients will be invited to Visit 2 at discharge or 14 days post-surgery at the latest, and to Visit 4 at 6 months.

### Data to be collected

**Fig 2:**
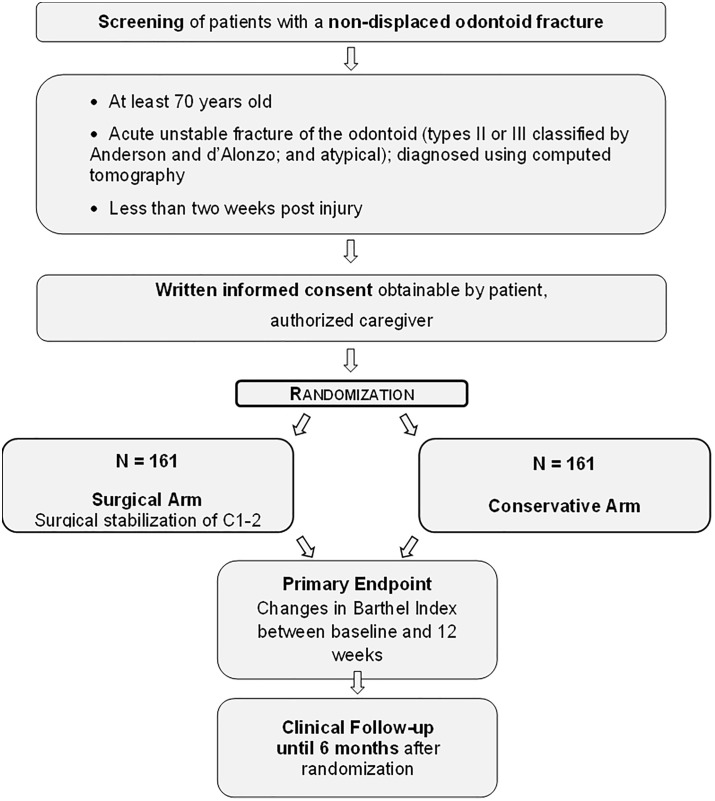
Study Flow Chart.

The frequency and scope of study visits are detailed in Fig 1. The Study Flow Chart is depicted in Fig 2.

Inclusion/Exclusion Criteria: Criteria determining whether a patient can participate in the study.

Informed Consent: The patient will be asked to give their written informed consent. In case they are unable to provide legal consent, i.e., due to cognitive impairment, an authorized caregiver will be asked to decide about study participation.

Baseline Characteristics: Initial patient data collected at baseline: age, sex, past medical and surgical history according to standard of care.

Charlson Comorbidity Index (CCI) (documented at baseline): A method to collate comorbidities of patients into a score that categorizes their overall severity. Each condition is assigned a score of 1, 2, 3 or 6, depending on the risk of dying associated with each one. Additionally, age is incorporated as a risk factor: patients receive 1 additional point for each decade over 50 years of age (i.e., 50–59 years = +1, 60–69 = +2, 70–79 = +3, ≥ 80 = +4).

### Recruitment status

Enrolment is anticipated to begin in the 3^rd^ quarter of 2025. We estimate recruitment completion (last patient in) by late 2027, given the enrolment target of 322 and the number of sites. The 6-month follow-up of the last patient should be completed by mid-2028. Thus, we expect the primary analysis to commence in Q3 2028, with results reported in late 2028. These timelines may be adjusted depending on actual recruitment rates.

Neurological Exam: Assessment of the patient’s neurological status to screen for neurological deficits caused by spinal cord injury from the odontoid fracture or other pathologies, according to standard of care.

Randomization: Patients will be randomized to either the conservative or surgical group according to a predefined process.

CT Cervical Spine: A computed tomography (CT) of the cervical spine will be obtained at the baseline visit to determine the fracture type and its dislocation measured in mm, and at Visits 3 to check for the presence of fracture healing (both groups), secondary dislocation in mm (both groups) and integrity of the implant material (surgical group).

Barthel Index (BI): A scale to measure performance in activities of daily living that constitutes the primary outcome in the statistical analysis after 12 weeks.

EuroQol-5D Questionnaire (EQ-5D): A standardized instrument to measure health outcomes, used within secondary analyses to compare the quality of life between groups.

VAS Neck Pain: A standardized instrument for assessing neck pain on a continuous, visually coded scale that is used in routine clinical practice.

Neck Disability Index (NDI)): A questionnaire used to measure neck-specific disability for both groups, constituting the secondary outcome.

Concomitant medications: Categorized by substance class; antidiabetics, anticoagulants, antithrombotic medicine, antiosteoporotic agents (vitamin D3, calcium, bisphosphonates), medication for movement disorders (e.g. Parkinson’s disease, etc.), antidepressants, steroids, and “other”.

Adverse Events (AEs): Monitoring of all AEs and documentation at each visit, with particular attention to safety-relevant adverse events of special interest (AESIs) and serious adverse events (SAEs).

Duration of Surgery: Time taken to complete the surgery (surgical group or cross-over).

Patient or caregiver diary: diary for patients to document daily duration of cervical collar wearing (conservative group).

Readmission to Hospital: Recording if the patient is readmitted to the hospital any time during the study until week 12, i.e., for the management of medical or surgical complications of either treatment.

Crossover to Surgical Group (see crossover visit): Noting if the patient switches from the conservative to the surgical arm due to failure of conservative treatment until week 12. In case of crossover and conduction of surgery, documentation of visits 1 (surgery), 2 (discharge) and 4 (6 months), after which the study concludes for the crossed patient.

### Overall duration of the study

The first patient will be included approximately in the 2^nd^ quarter of 2025 and the last patient approximately in the 4^th^ quarter of 2027. Accordingly, the last patient will exit the study (‘last patient last visit’) approximately 2^nd^ quarter 2028.

### Randomization

After giving informed consent, patients are randomized to study arms during the baseline visit using randomizer.at, an online-randomization tool. Randomizer.at uses pre-defined randomization algorithms which are tailored to our study needs. Allocation occurs on demand in a 1:1 ratio, stratified by study center using permuted blocks. Assignment to the study arm is concealed and only revealed at the time of randomization through the password-protected, secure website.

### Sample size calculation

Assuming a non-inferiority study design with a one-sided α = 0.025, we set the expected difference in change of BI scores between the two groups to 0. Unnanuntana et al. (2018) show that the minimal clinically important difference (MCID) of the BI in elderly patients after a surgical intervention is 9.8 [48]. Bouwstra et al. (2018), conclude the smallest detectable change in BI in a geriatric nursing home population to be 3.0 [32]. It is common practice to choose a non-inferiority margin for a patient-reported outcome such as the BI, which is lower than the MCID, see Copay et al [49]. Thus, we set the non-inferiority margin to 5, which is closer to the smallest detectable change than to the MCID and therefore more conservative. The standard deviation of the difference in BI between baseline and week 12 was estimated at 12.7 referencing prior work by Unnanuntana et al [48]. Based on these assumptions, a sample size of 137 in each group (274 in total) will have 90% power to reject the null hypothesis of the one-sided 2.5% significance level t-test that the surgical group is inferior to the conservative group in favor of the alternative hypothesis that the surgical group is non-inferior to the conservative group. Assuming a drop-out rate of 15%, a total of 322 patients will need to be included (161 per arm).

### Statistical analyses

Analysis will be performed on all patients who were randomized to the study. The primary estimand and its attributes are described in Table 1.

**Table 1:** Definition of all five attributes used to construct the primary estimand for the SCORE study (non-inferiority setting).

Primary estimand	
**Population**	Geriatric patients with recent type II & III odontoid fractures (as defined by trial inclusion/exclusion criteria).
**Variable**	Functional status accessed as the change in Barthel Index (BI) between baseline and 12 weeks thereafter
**Treatment**	• Surgical stabilization C1-2 by dorsal screw-rod construct (duration of treatment: Approximately 100 min + approximately 6 days hospital stay) or • Conservative therapy by external cervical collar immobilization (duration of treatment: 12 weeks)
**Population-summary measure**	Mean difference, including 95%CI.
**Intercurrent event (IE) and strategy**	• Refusal of therapy as randomized – multiple imputations (hypothetical estimate) • Crossover from the conservative to the surgical arm after randomization but before week 12 – use the last available BI value before surgery as best estimate of the hypothetical BI value at week 12 *Note: Cross-over from the conservative to the surgical arm after week 12 is allowed, as it does not influence the primary variable, which is measured at week 12* • Loss to follow-up after start of therapy – multiple imputations; missing at random assumed • Death – (expected ca. 10%) multiple imputations (hypothetical estimate)

Intercurrent events (IEs) will be dealt with using a hypothetical strategy to determine the treatment effect without the abovementioned IEs.

The primary endpoint will be tested using a mixed model for repeated measures (MMRM) of BI, where treatment, time, their interaction, baseline BI scores, age, and CCI will be fixed covariates, and study center will be a random covariate. In case the model does not converge due to small centers, the covariate center will be abandoned. The adjusted mean difference between groups in the primary outcome will be tested at the 12-week time point.

### Research ethics approval and consent

This study protocol (version 1.1 dated 12 March 2025) has been approved by the Ethics Committee of the lead study site (Technical University of Munich) with the reference number 2024–630-S-NP. In addition, each participating site’s local Ethics Committee has reviewed and approved the protocol prior to initiating the study at that site or has accepted the lead site’s approval in a multi-center framework. No patients will be enrolled at a site until that site has obtained the necessary ethical approval.

All participants (or their legal representatives) provide written informed consent before any study procedures. The consent form describes the study interventions, that participants will be randomized to one of two standard treatments, and outlines potential risks/benefits. It emphasizes that participants are free to withdraw at any time. Patients are informed that their pseudonymized data will be collected, stored, and used for research, and that monitors/auditors may review their records. Consent for publication of results is included in the consent.

analyses will be applied to the secondary outcomes with pairwise between-group comparisons at 12 weeks and 6 months. The estimated mean and standard error and the 95% confidence interval (CI) for the mean treatment effect will be reported for each follow-up time point. Differences between surgical and non-operative treatments will be assessed by estimating the main effects of treatment as well as time-and-treatment interactions. For binary outcomes generalized estimating equations will be used. Treatment effects will be estimated as differences in estimated proportions. Each estimated outcome score will be plotted by treatment group to illustrate change over time. No adjustments will be made for multiple comparisons.

Sensitivity analysis of the primary endpoint will be done by repeating the primary endpoint analysis “as treated” by counting each patient who was treated surgically in the surgical group, regardless of whether they were assigned to the surgical group or crossed over at a later time point.

Adverse events will be coded with MedDRA prior to analysis.

Primary and secondary analyses will use all available data at the pre-determined time points. The extent of missing data will be calculated at each time-point. Patients with missing data at the 3-month time point will be compared to those who participated in all visits. Variables asso-ciated with missing data and baseline characteristics will be included as covariates in the mixed model as a further sensitivity analysis of the primary endpoint. Additionally, multiple im-putations will be used on data for each outcome measure at each time point. The results will be presented as adjusted mean or percentage and treatment effect including 95% CI from the 10 iterations.

No interim analysis for efficacy is planned, so the study will continue until full enrolment is reached or unless stopped early for safety or futility by recommendation of the SMB.

### Organizational aspects

Organizational project- and data management, as well as monitoring is performed by the Münchner Studienzentrum (MSZ), the independent clinical research center of the Technical University of Munich, School of Medicine and Health. For advice on safety aspects, an independent safety monitoring board (SMB) with a SMB Charta has been established. The patient’s perspective is represented by three patient organisations (BfO, LfO, OSD) which participate in the study’s Trial Advisory Board. The study is performed in adherence to the Principles of the Declaration of Helsinki, applicable data protection law and the clinical study protocol. Data will be documented and stored in a validated study database (Macro). All sites agreed to adhere to the instructions and procedures described in the study protocol. The most recent version of the study protocol is available at http://www.clinicaltrial.gov.NCT06961578.

## Discussion

### Rationale

There exists considerable controversy about the treatment of unstable geriatric OFs (most commonly Anderson & d’Alonzo types II and III as well as atypical C2 fractures) [6,13,39,5,50]. While for displaced fractures with neurological injury conservative treatment is unequivocally considered contraindicated, there is uncertainty as to whether this particularly vulnerable patient population should be subjected to a surgical intervention under general anesthesia or prolonged conservative treatment, each posing their own risks and hazards. The population of the elderly is at an increased risk for prolonged morbidity, functional disability and impaired quality of life after injury, which led to the decision of setting the minimum age to 70 years for inclusion [2,5,28,51].

Due to the rising prevalence as well as the clinical and socioeconomical impact of OFs, Level I evidence from a randomized clinical trial regarding optimal treatment would provide a clear clinical utility for any health-care provider world-wide. Both treatment options – immobilization via a cervical collar and surgical stabilization with a screw-rod-system – are established procedures requiring standard materials only. Overall, there is a slight trend in favour of surgical therapy based on expert opinions and retrospective studies [14]. However, no large multi-center study has yet been carried out that compares conservative and surgical therapy of geriatric OFs randomized. There are two yet unpublished clinical trials: INNOVATE and USOFT, which are enlisted within trial registries [24,26]. These trials however put emphasis on a different scientific hypothesis; the main focus of the planned clinical study lies in the quality of life and functional autonomy of elderly patients, which should represent the prime outcome benchmark for a specialist treating this population. Geriatric patients are especially vulnerable to acute injuries that may kickstart a cascade of further severe comorbidities [7,21,52]. By optimizing and standardizing the therapy of OFs, a number of serious consequences can be prevented [15,53,54]. In the same vein, clinical dementia rates in the elderly population reach up to 15%, which entails dependency on nursing homes [14]. This confounds the success rate of the conservative treatment, causing its failure in 50–60% due to non-compliance in wearing the collar without supportive nursing care [17]. We deliberately designed both the inclusion and exclusion criteria to mirror real-life practice, including patients with dementia specifically because of their expected non-compliance and associated complications prominent in this subpopulation. Thus, we aim to provide high-class evidence that is transferrable to clinical practice and guideline recommendations.

Lastly, the socioeconomical burden of an aging population with multiplying comorbidities cannot be understated. Geriatric patients failing to recover from OF may cause huge expenses for readmissions and delayed surgery, requiring a far more extensive follow-up, outpatient resources, and appliances [7,11,12,28,35,40,51,55]. It is therefore of utmost importance to forestall these expenses by optimizing the therapy of OFs and reducing hospital-associated disabilities, complications and long-term deterioration of elderly patients [7,19,35,52]. A common verdict of recent publications is the need for a prospective trial comparing surgical and conservative strategies [15,46,56]. In this respect, the planned clinical study exhibits significant potential to relieve the burden of disease for these vulnerable patients by producing high quality data. This bears particular significance considering the drastic lack of scientific evidence existing for the geriatric population [37].

### Aims and outcomes of the trial

The principal goal is to assess the functional autonomy of elderly patients. The BI, originally described in 1955, is a 10-item measure of activities of daily living and has been proven to be reliable and precise especially in the elderly [30–36]. It is commonly used as a functional outcome indicator and predictor of disability after traumatic events such as hip fractures [31,33,48]. We specifically chose 12 weeks for the primary outcome to mitigate the rates of loss of follow up and compliance, which is a known issue in this patient population [7,14,15,21,23,44]. To assess the quality of life we will additionally employ the EuroQol 5D. An instrument designed for the disability of patients with conditions of the cervical spine is found in the neck disability index and VAS for chronic neck pain [29]. These instruments are all validated in literature and widely adopted for scientific problems of similar nature [57,58]. The radiographic outcome encompasses the assessment of bony fusion on CT scans obtained at follow-up, which represents standard of care. An important assessor of patient compliance will be the time of wearing the cervical collar per day in hours, which will be recorded in a patient diary by the patients themselves or their authorized caregiver. The number of patients undergoing surgery despite conservative therapy (cross-over rate) will be captured. Adverse events represent a key outcome assessor for both arms, particularly in the in-hospital setting, all adverse events will be assessed during study participation.

## Supporting information

S1 Checklist SPIRIT 2025 editable checklist. (DOCX)

## Abbreviations

AE: adverse event
ASA: American society of anesthesiologists
BI: barthel index
BMFTR: bundesministerium für forschung, Technologie und Raumfahrt (German Federal Ministry of Research, Technology and Space)
CCI: charlson comorbidity index
CI: confidence interval
CT: computed tomography
eCRF: electronic case report form
EQ-5D: EuroQol 5-dimension questionnaire
GDPR: general data protection regulation
IE: intercurrent event
IRB: institutional review board
MSZ: münchner studienzentrum
NDI: neck disability index
OF: odontoid fracture
PI: principal investigator
PP: per protocol
SAE: serious adverse event
SMB: safety monitoring board
SPIRIT: standard protocol items: recommendations for interventional trials
SCORE: surgical versus conservative treatment of odontoid fractures in the elderly
TUM: Technical University of Munich
VAS: visual analog scale
